# Development and evaluation of gluten free pasta from brown rice, quinoa, and chickpeas for celiac disease patients

**DOI:** 10.1038/s41598-025-06516-6

**Published:** 2025-06-20

**Authors:** Ahmed M.S. Hussein, Sayed Mostafa, Naglaa A. Shedeed, Mohie M. Kamil, Mona Y. Mostafa, Nefisa A. Hegazy

**Affiliations:** 1 Department of Food Technology, Food Industries and Nutrition Research Institute, National Research Centre, El-Buhouth Street, Dokki, Cairo, 12622 Egypt; 2 Home Economics Department, Faculty of Specific Education, Mansoura University, Elgomhouria Street, Mansoura City, 35516 Egypt

**Keywords:** Chemical analysis, Cooking quality, Physical properties, Organoleptic properties and gluten-free pasta, Celiac patients, Biochemistry, Materials science

## Abstract

The demand for gluten-free products for people suffering from a gluten allergy is increasing. Therefore, the aim of this study was to produce high-quality gluten-free pasta from brown rice flour (BRF), quinoa flour (QF) and chickpea flour (CPF), semolina flour (S) was used as control sample. The chemical composition of the raw materials showed the higher protein, carbohydrate and fat content of CPF, S and QF, respectively. In addition, the content of Ca, K, and Fe was higher in CPF. The total concentration of essential amino acids in CPF, BRF and QF was between 38.9 and 34.04%. Pasta was prepared with different levels of CPF, BRF, and QF. The quality of the pasta was evaluated chemically and physically. The chemical analysis showed that the formula (BRQ5) had the highest protein, fat, ash and fiber content. The color analysis showed that the darkness of the pasta increased with increasing QF. The overall acceptability of the different pasta showed that the control (S) had the highest acceptability, followed by BR and BRQ1. The analysis of the texture profile showed that the hardness of the uncooked control pasta was the highest. In summary, it can be recommended to produce gluten-free pasta with the BRQ1 formula.

## Introduction

Pasta is one of the most popular dishes in the world because it is easy to prepare, tasty, long-lasting shelf life, and inexpensive. In the last ten years, its production has increased from 7 to 12 million tons annually^1–4^. The high gluten content of durum wheat flour, which is mainly responsible for the formation of the matrix structure, gives it special properties such as reduced cooking loss, high hardness, stickiness, and tolerance to overcooking^5^. There is an increasing demand for gluten-free foods for people with gluten allergies (e.g., celiac disease)^6,7^. The gluten-free products on the market are based on starch, mainly from rice and maize, which produces goods with low nutritional value. The technological improvement of gluten-free pasta aims to use proteins such as eggs, pseudocereals, legumes, starch, and hydrocolloids (gum) to reproduce/improve the gluten network of wheat^8^. There is a lack of protein, fiber, minerals (calcium, magnesium, iron, and zinc), and vitamins (folate, niacin, vitamin B12, and riboflavin). Quinoa-based pasta is a good alternative as it has excellent nutritional value and contains essential amino acids and bioactive elements (such as vitamins like folate, tocopherols, and flavonoids like kaempferol and quercetin)^9,10^.

Many studies have looked at how to make pasta without the gluten network. To improve the quality of gluten-free pasta, a number of additives and texturizing chemicals are routinely used, including protein isolates, hydrocolloids, emulsifiers, and modified starches^11^. Prior to starch gelatinization and subsequent retrogradation, dough heating and cooling procedures are the mainstays of gluten-free technology^12^.

Brown rice contains a wide range of functional and nutritional components, including dietary fiber, gamma-aminobutyric acid, phytosterols, minerals, lipids, amino acids, vitamin E, and phenolic compounds^13^. Furthermore, brown rice has more nutrients than milled white rice, as only the husk, i.e. the outermost layer, is removed during the milling process. Moreover, several studies have documented positive health effects associated with the consumption of brown rice. Brown rice can help lower blood pressure in middle-aged men and women and may also be an excellent way to control body weight^14^. According to a study by Sun^15^, brown rice may reduce the incidence of type 2 diabetes. Therefore, the main focus of research on brown rice has been on its physiological benefits and functional elements.

Quinoa protein has a balanced ratio of amino acids, including relatively high amounts of lysine (5.1–6.4%) and methionine (0.4–1.0%). Quinoa proteins are a source of bioactive peptides. Potentially bioactive peptides could be used as new ingredients in the development of nutraceuticals or functional foods to reduce oxidative stress-related diseases such as cancer^16^. Unfortunately, the outer layers of the quinoa seed coat contain bitter-tasting compounds (water-soluble saponins) that can have a bitter effect on food. For this reason, the coating of most commercially consumed quinoa seeds is removed via water washing and milling to remove anti-nutritional compounds before consumption^17^. The World Health Organization (WHO) has evaluated quinoa as comparable to milk in terms of nutrition due to its high content of riboflavin, potassium, B6, thiamin, and niacin, as well as zinc, magnesium, manganese, and copper.

The chickpea is a significant food legume and an important part of many people’s diet. After the common bean (*Phaseolus vulgaris L*.) and the pea (*Pisum sativum L.)*, the chickpea (Cicer arietinum L.) is the third most important edible legume grown worldwide,11. It is known to be an excellent source of dietary fiber, protein, vitamins, and minerals and is an important part of the daily diet in many countries^18,19^. The health benefits of eating chickpeas include reducing the risk of diabetes, cardiovascular disease and cancer^19^. Among edible legumes, chickpea is the hypocholesterolemic agent^20^.

This study aimed to resolve the lacks of quantity and quality of gluten free product, where recently celiac disease increased and local market needs more gluten free products. Pasta is one of the most spread and daily intake food products. Therefore, brown rice flour (BRF), quinoa flour (QF) and chickpea flour (CPF) were selected and evaluated for their gluten-free properties and their high nutritional value to produce gluten free pasta.

## Materials and methods

### Materials

Semolina was obtained from the North Cairo Flour Mills Company, Egypt. Brown Rice (*Oryza Sativa*), Quinoa seeds (*Chenopodium quinoa Willd*.), and Chickpeas (*Cicer arietinum kabuli*) were purchased from a local market in Giza, Egypt. Carboxy methyl cellulose and Glycerol monostearate were procured from SIGMA Chemicals, St. Louis, Missouri, United States. petroleum ether Merck, Darmstadt, Germany. All other chemicals used were of analytical grade.

### Methods

#### Preparation of Raw materials

Chickpea flour and rice flour were prepared according to Prasad^21^ as follows: Chickpea grains and rice were sorted separately to remove extraneous contaminants such as stones, damaged and discolored grains, and sticks. Then, they were ground using a small Quadrumat mill (Brabender made in Germany), and the flour was obtained after sieving through 40- mesh. Each type of flour was subsequently packed in polyethylene bags until use.

Quinoa seeds were cleaned to eliminate undesired materials such as mud, dirt, stone, dust, and leaves and then soaked in water for 2 h at room temperature to remove saponin materials. Quinoa seeds were washed with tap water until the froth disappeared from the water, and then dried at 50 °C for 12 h to eliminate saponin. To make the full meal, the quinoa seeds were ground to a fine powder in small Quadrumat mill (Brabender made in Germany).

#### Rheological properties of the dough

Rheological properties of different pasta formulas were evaluated via the following methods:

### Rapid visco analyzer (RVA)

The RVA was determined via different parameters of Rheolab QC, Anton Paar, made in Austria, in the Food Technology Department, National Research Centre. Accordance^22^.

Flour formulas samples were weighed 3.5 g on the basis of 14% moisture and then dispersed in 25 ml of distilled water for the RVA measurement. Suspensions were held at 50 °C for 1 min and then raised to 95 °C at a rate of 12 °C/min, held at 95 °C for 2.5 min and cooled to 50 °C at the same rate, and finally held at 50 °C for 2 min. The stirring rate was 960 rpm for the first 10 s followed by 160 rpm for the remaining test. Peak Vis. (cP) breakdown (cP), final viscosity (cP), Setback (cP) Peak time (min), pasting temperature (°C), Peak temperature (°C) were obtained from the pasting curve.

#### Gross chemical composition

The gross chemical composition of the different samples was determined.The moisture content and the chemical composition of the samples were determined by drying performed according to the AACC Approved Methods (2000)^22^. In addition, the total protein content (AACC, Method 46 − 08), the ash content (AACC, Method 08 − 01) and the fat content (AACC, Method 30 − 26; using hexane solvent) of the samples were determined.

The total carbohydrate content was determined as follows: total carbohydrate content = 100 – (protein + ash + fat + crude fiber). Moreover, total calories were calculated according^23^ via the following equation:

$$\:\text{T}\text{o}\text{t}\text{a}\text{l}\:\text{c}\text{a}\text{l}\text{o}\text{r}\text{i}\text{e}\text{s}\hspace{0.17em}=\hspace{0.17em}\text{F}\text{a}\text{t}\:\times\:\:9\hspace{0.17em}+\hspace{0.17em}\text{P}\text{r}\text{o}\text{t}\text{e}\text{i}\text{n}\:\times\:\:4\hspace{0.17em}+\hspace{0.17em}\text{A}\text{v}\text{a}\text{i}\text{l}\text{a}\text{b}\text{l}\text{e}\:\text{c}\text{a}\text{r}\text{b}\text{o}\text{h}\text{y}\text{d}\text{r}\text{a}\text{t}\text{e}\:\times\:\:4$$

### Mineral

The mineral contents of semolina, brown rice, quinoa and chickpea were determined by using flame photometry method for sodium and potassium, and flame atomic absorption spectrometry for calcium, iron, phosphorus and zinc^24^.

#### Amino acids

The method was used to assess the amino acid profile of different flours (semolina, brown rice, quinoa and chickpea) on a dry weight basis; amino acids were represented as g/100 g protein and were evaluated according to the method of^25^.

The chemical score (CS) was calculated according to the FAO/WHO. mg of essential amino acids in g test protein C. S mg of essential amino acids in the requirement pattern.

The computed protein efficiency ratio (C-PER) C-PER was calculated^26^ with the following equation:

$$\:\text{C}-\text{P}\text{E}\text{R}\hspace{0.17em}=\hspace{0.17em}0.684\hspace{0.17em}+\hspace{0.17em}0.456\:\left(\text{L}\text{e}\text{u}\text{c}\text{i}\text{n}\text{e}\right)\:-\hspace{0.17em}0.047\:\left(\text{p}\text{r}\text{o}\text{l}\text{i}\text{n}\text{e}\right).$$

The computed biological value (BV) Biological value was calculated^27^ according to the following equation:

$$\:\text{C}\text{o}\text{m}\text{p}\text{u}\text{t}\text{e}\text{d}\:\text{B}\text{i}\text{o}\text{l}\text{o}\text{g}\text{i}\text{c}\text{a}\text{l}\:\text{V}\text{a}\text{l}\text{u}\text{e}\:\left(\text{B}\text{V}\right)\hspace{0.17em}=\hspace{0.17em}49.9\hspace{0.17em}+\hspace{0.17em}10.53\text{C}-\text{P}\text{E}\text{R}$$

A/E ratio: essential amino acid content in the food protein (A)/total essential amino acid content (E) ratio; PER: protein efficiency ratio; B.V.: biological value.

### Pasta Preparation

A standard pasta sample was prepared from semolina flour as described previously^22^. The flour sample was mixed and formed via a lab pasta maker (Matic 1000 Simac Machine Corporation, Millano, Italy) at the Food Technology Department, National Research Centre. The modified formula used to prepare the free gluten pasta is clearly shown in Table (1). Moreover, 1% glycerol monostearate (GMS) and 2% carboxy methyl cellulose were used to improve the textural properties of gluten-free pasta. Pasta was dehydrated for 15 min in ambient air and dried for 10 h in a cabinet dryer at 70°C^28^. The sample was subsequently cooled to room temperature, packaged in polyethylene bags, and stored until use.

**Table 1:** Formulas of gluten-free prepared pasta.

Sample Code	Semolina (S) %	Brown rice flour (BR)%	Quinoa flour (QF) %	Chickpea flour (CPF)%	Glycerol monostearate (%)	Carboxy methyl cellulose (%)
Control	100	-	-	-	-	**-**
BR	-	100	-	-	1	2
BRQ1	-	80	10	10	1	2
BRQ2	-	70	20	10	1	2
BRQ3	-	60	30	10	1	2
BRQ4	-	50	40	10	1	2
BRQ5	-	40	50	10	1	2

BR:100% Brown Rice; BRQ1: 90% Brown Rice with 10% quinoa flour; BRQ2: 80% Brown Rice with 20% quinoa flour; BRQ3: 70% Brown Rice with 30% quinoa flour; BRQ4: 60% Brown Rice with 40% quinoa flour; BRQ5: 50%Brown Rice with 50% quinoa flour.

### Cooking quality

Cooking quality was determined via weight increase, volume, and cooking loss^22^.

Cooking loss (%) was measured as the amount of cooking water evaporated to dryness, using the oven-dry method (AACC, Method 44–15 A)^22^.

### Color

Pasta color was determined by using Hunter, Lab Scan XE-Reston VA, USA. The color value is expressed digitally through L* whiteness (black to white, 0 −100, a* (positive = redness, greenness = negative) and b* (positive = yellowness, negative = blueness).

### Sensory evaluation of pasta

Quantitative descriptive analysis was used to measure the sensory attributes of gluten free pasta samples prepared with different flour. Twenty trained people(15 male + 5 females) at the Laboratory of Food Technology, National Research Centre, evaluated the quality of the sensory parameters. Each pasta sample was cooked separately in boiling distilled water for 8 min. After cooking, the pasta was directly drained, coded and served (65 and 70 °C) in randomized order. Panelists have evaluated products for color, flavor, appearance, tenderness, stickness and general acceptability^28^.

### Textural profile analysis (TPA)

The texture of the pasta was evaluated before and after cooking using instrument A texture analyser, (Brookfield model-CT3-10 kg) mad in (USA). The dimensions of the uncooked pasta samples were 25 mm in length and 8 mm in diameter. The test methods for uncooked pasta were as follows: fixture TA-SBA, test type rupture, correction 7 N, trigger load 7 N, and test speed 2 mm/s. double compression test to penetrate to 50% depth, at 1 mm/s speed test. While texture of pasta after cooking was evaluated (TPA), the trigger load to reach the target distance was 9 N, and the test speed was 2 mm/s. The dimensions of the uncooked pasta were 36 mm in length and 13 mm in diameter. The test method was carried out as follows: Test type was TPA, probe TA. AACC36, trigger load 9 N, Target 3 mm, test speed 2 mm/min.

### Statistical analysis

Statistical analysis (means ± SDs) of three replicates for each experiment are statistically analyzed using one-way analysis of variance (ANOVA), and Duncan’s test were used to obtain the SE. SAS Systems for Windows software, mean version 6.12 TS020 (SAS, Statistical Analysis System, Institute Inc., Cary, NC, 1996), was used to statistically analyze all pasta and raw material findings. We employed analysis of variance (ANOVA) and the least significant difference (LSD) test (P˂ 0.05) to find significant differences between the treatment means.

## Results and discussion

### Chemical composition of the tested flour

Brown rice (BRF), quinoa (QF) and chickpea flour (CPF) were selected to produce gluten-free pasta. The gluten free flour samples were chemically compared with the semolina flour samples (control pasta flour) as shown in Table (2). The moisture content of all flour samples ranged between 11.53 and 13.28%. CPF was characterized by higher protein (23.19%) and fat (6.04%) content than semolina (protein 8.60% & fat 1.22%). The lowest total carbohydrate content was found in QF and CPF, where they declined to 57.15 and 52.71%, respectively. The results of semolina flour agreed with^29,30^. The higher fat content was found in QF (6.07%) and CPF (6.04%), while the fat content in BRF and semolina decreased to 2.3 and 1.22%, respectively. In addition, QF and CPF contained higher levels of fiber than BRF and semolina. These results were in agreement with those of^31,32^ which reported that rice flour contained 6.65% protein, 0.38% crude fiber, 0.54% ash, and 83.33% carbohydrate. In addition^33^, it was reported that rice flour contained 7.80% crude protein, 0.85% ash, 0.45% fiber, and 90.20% available carbohydrate. Furthermore, several researchers have evaluated the chemical composition of previously selected free gluten meal, i.e.,^34–37^.

In addition to the chemical composition, the total caloric content was also calculated to determine the expected calorie content of the selected gluten-free flour. The results showed that the total energy content of the gluten-free flour tested ranged between 357 and 339.71%.

**Table 2:** Gross chemical constituents of the tested flour.

parameter	Semolina	BRF	QF	CPF	L.S.D at 0.05
Moisture (%)	11.65^b^ ± 0.11	12.01^ab^ ± 0.17	13.28^a^ ± 0.29	11.53^b^ ± 0.28	0.73
Protein (%)	10.90^c^ ± 0.19	8.60^d^ ± 0.27	14.12^b^ ± 0.25	23.19^a^ ± 0.48	0.06
Fat (%)	1.22^c^ ± 0.05	2.30^b^ ± 0.05	6.07^a^ ± 0.03	6.04^a^ ± 0.07	0.07
Ash (%)	0.79^d^ ± 0.02	1.25^c^ ± 0.07	2.38^b^ ± 0.01	2.48^a^ ± 0.03	0.07
Fiber (%)	0.81^d^ ± 0.03	1.49^c^ ± 0.32	7.0^a^ ± 0.37	4.05^b^ ± 0.02	0.34
T. Carbohydrate (%)	74.63^a^ ± 0.72	74.35^b^ ± 0.56	57.15^c^ ± 0.85	52.71^d^ ± 0.75	0.09
Total calories K Cal	353.1	353.22	339.71	357.96	

* Semolina (S). Brown rice flour (BRF). Quinoa flour (QF). Chickpea flour (CPF), the obtained results represent the mean value *n* = 3 ± SD. The same letter in a row is not significantly different.

### Mineral contents of the tested flour

Table 3 displays the mineral composition of chickpea flour, quinoa flour, brown rice flour, and semolina flour. According to the findings, chickpea flour has the greatest concentrations of calcium, potassium, and iron, followed by quinoa flour and semolina flour. Among the various flours, OF had the greatest phosphorus concentration (457 mg/100 g). Brown rice flour has a greater zinc level than the other chosen flours. Iron (1.47 mg/100 g), phosphorus (33.3 mg/100 g), magnesium (143 mg/100 g), potassium (223 mg/100 g), sodium (7 mg/100 g), zinc (20.2 mg/100 g), and calcium (23 mg/100 g) are all found in different amounts in brown rice flour, according to^38^ other studies. Quinoa is high in minerals, such as calcium, iron, zinc, magnesium, and manganese, which give the grains high value^39 ^and chickpea flour^40^.

**Table 3:** Mineral contents of the tested flour.

Minerals (mg/100 g).	S	BRF	QF	CPF	LSD at 0.05
Calcium	50.89^b^ ± 0.11	31^c^ ± 0.09	47^b^ ± 0.13	105^a^ ± 0.17	4.71
Phosphor	120^c^ ± 0.15	32.4^d^ ± 0.19	457^a^ ± 1.12	366^b^ ± 0.82	5.35
Potassium	205.33^c^ ± 0.52	182^d^ ± 0.33	563^b^ ± 0.65	875^a^ ± 2.13	5.07
Sodium	630.18^a^ ± 0.52	22.9^b^ ± 0.13	5^c^ ± 0.03	24^b^ ± 0.05	4.59
Iron	2.35^c^ ± 0.05	1.6^d^ ± 0.01	4.57^b^ ± 0.05	6.24^a^ ± 0.09	0.23
Zinc	3.36^b^ ± 0.03	12.15^a^ ± 0.10	3.1^b^ ± 0.07	3.43^b^ ± 0.03	0.28

Semolina (S). Brown rice flour (BRF). Quinoa flour (QF). Chickpea flour (CPF), values are the means ± SDs (*n* = 3). a-d Means in the same row with different superscripts are significantly different.

#### Total amino acid contents of semolina, brown rice, Quinoa and Chickpea flour

The amino acids content of foods plays an important role in assessing their nutritional value. Therefore, the amino acids of selected gluten-free flour samples were determined and are presented in Table 4. The results indicated that the total essential amino acid content (29.95 g/100 g) of the semolina flour was lower than that of the selected gluten-free flour. The total essential amino acid content was highest in chickpea (38.9 g/100 g) and brown rice (38.1 g/100 g), followed by quinoa (34.04 g/100 g). Leucine was the first limiting amino acid in chickpea flour (8.70 g/100 g), and lysine was the second limiting amino acid (7.20 g/100 g). Moreover, the biological value (BV) was highest in chickpea flour, followed by brown rice flour, quinoa flour, and semolina, where it reached 97, 90.13, 84.89, and 77.29%, respectively. The calculated protein efficiency ratio (C-PER) also showed the same trend as BV^41^.

**Table 4:** Amino acids composition of the tested flour.

Amino acids	S	BRF	QF	CPF
Essential amino acids (NEAA) g/100 g protein				
Tryptophan (TRP)	1.14	0.15	1.10	0.9
Therionine(THR)	3.36	4.71	3.35	3.1
Valine(VAL)	2.85	6.60	4.40	4.6
Methionine	1.33	1.72	2.75	1.1
Isoleucine(ILE)	3.90	5.63	3.55	4.8
Leucine(LEU)	5.15	7.39	6.20	8.7
Phenylalanine(PHE)	4.22	5.54	2.75	5.5
Hisitidine(HIS)	2.33	2.84	5.90	3.0
Lysine(LYS)	2.02	3.52	4.0	7.2
Total EAA	29.95	38.1	34.04	38.9
Nonessential amino acids(NEAA) g/100 g protein				
Serine(SER)	4.95	3.91	4.70	3.7
Glutamic(GLU)	28.11	13.85	14.30	17.3
Proline(PRO)	9.22	4.95	4.00	3.8
Glycine(GLY)	3. 37	5.51	5.50	3.7
Alanine(ALA)	3.38	5.30	4.45	4.8
Aspartic (ASP)	10.02	10.51	9.50	11.0
Tyrosine(TYR)	2.33	3.20	2.65	3.0
Argnine(ARG)	4.6	8.30	8.60	8.3
Cystine(CYS)	1.11	1.50	2.15	0.60
Total NEAA	73.80	57.03	55.85	56.2
Computed B.V. (%)	77.26	90.13	84.89	97.00
C-PER (g)	2.59	3.82	3.32	4.47

Semolina (S). Brown rice flour (BRF). Quinoa flour (QF). Chickpea flour (CPF), C-PER = computed protein efficiency ratio calculated as follows: C-PER = 0.684 + 0.456 (Leucine)- 0.047(proline). BV = computed biological value was calculated via the following equation: BV = 49.9 + 10.53 C-PER

#### Pasting profile (RVA) of the Raw materials

The pasting properties of the Rapid Visco-Analyzer (RVA) for the gluten-free flour samples were determined and are presented in Table (5). Five formulations of mixtures of brown rice, quinoa, and chickpea flour were prepared (see Table 1) and compared with control samples of 100% semolina, and 100% brown rice. The peak viscosity gradually decreased with increasing the percentage of quinoa flour, with the highest peak viscosity reaching 3516 and 3462 cP in the semolina and brown rice control samples, respectively. However, the lowest peak viscosity decreased to 2620 cP in PRQ5. The same trend was observed for the parameters Trough, Break down, Final viscosity Setback and Pasting temperature. The peak viscosity obtained also indicates that brown rice, BRQ1 and PRQ2 are the best formulas compared to the control sample (semolina), where the percentage decrease in peak viscosity compared to the control sample was 1.5, 2.1, and 13.9%, respectively. The results obtained are in agreement with^42^ Gómez, who found that peak viscosity, breakdown, and setback decreased when chickpea flour was used instead of wheat flour. The low carbohydrate content and the different composition of carbohydrates and proteins in chickpea flour probably influence the viscosity measurements^43^. The pasting temperature may be related to the ability of the material to bind water. The lowest peak, trough, breakdown, final and restoring viscosities and pasting temperatures were found in flour blends, according to the RVA properties of the flour samples^44,45^. As gluten-free flour was used instead of regular flour, the amylose/amylopectin ratios of the different starches changed, and a decrease in gluten content was observed^46^. Decrease in peak viscosity (cP), final viscosity, breakdown and rebound, indicating severe breakage of the amylose chain and loss of retrograde ability. This can be attributed to molecular degradation^46^.

**Table 5:** RVA parameters of blends from semolina control, brown rice BR, Quinoa flour and Chickpea flour.

parameter	Control	BR	BRQ1	BRQ2	BRQ3	BRQ4	BRQ5
Peak Vis. (cP)	3516	3462	3389	3027	2890	2750	2620
Decline (%)	---	1.5	2.1	13.9	17.8	21.8	25.5
Trough1 (cP)	1470	1150	1121	840	795	750	680
Break down (cP)	1700	1770	1890	1742	1650	1580	1470
Final Vis. (cP)	3399	3185	3047	2882	2750	2630	2410
Setback (cP)	458	445	342	330	305	280	265
Peak Time)min)	12.0	10.8	10.9	10.7	10.10	11.5	10.9
Pasting Temp. (°C)	60.2	64.5	62.0	61.6	60.0	58.5	56.5
Peak Temp. (°C)	94.8	94.7	94.7	94.7	93.5	92.0	89.5

Decline % = Decline Percentage of Peak Viscosity compared to control sample (Semolina). Control: 100% semolina flour; BR:100% Brown Rice; BRQ1: 90% Brown Rice with 10% quinoa flour; BRQ2: 80% Brown Rice with 20% quinoa flour; BRQ3: 70% Brown Rice with 30% quinoa flour; BRQ4: 60% Brown Rice with 40% quinoa flour; BRQ5: 50%Brown Rice with 50% quinoa flour.

#### Chemical composition of gluten-free pasta

The chemical compositions of pasta made with 100% semolina flour was selected as a control pasta sample, 100% brown rice flour (BRF) 100% for gluten free pasta, and mixtures of BRF and QF at different concentrations (10–50%) with 10% chickpea flour were prepared as treated gluten free pasta. The chemical quality of the studied samples is clearly shown in Table 6. Moisture content of control pasta and all free gluten pasta samples were ranged between 6.50 and 7.90%. This lower value is good for maximizing shelf life of pasta product^47^. While, Ruszkowska and Nowicka (2017)^48^ found that pasta moisture should not be excessively lower than 6% to avoid it become more brittle.

Also Table (6) showed that, mixing Quinoa flour with brown rice improved protein content of gluten free pasta compared to pasta 100% semolina and pasta 100% brown rice (control 1 and control 2). The same trend was noticed in other chemical contents except total carbohydrate. Whereas, pasta of 100% semolina contain fat ash and fibre reached to 1.05, 0.92 and 0.83%, respectively, while BRQ5 contained higher percentage from fat, ash and fibre reached to 4.50, 2.42 and 4.10, respectively. These results agreed with^47–50^.

**Table 6:** Proximate analysis (%) of gluten-free brown rice pasta formulated with Quinoa flour and Chickpea flour.

Samples	Moisture	Protein	Fat	Ash	Fiber	Carbohydrate	Total calories
Control	6.50^b^ ± 0.20	11.00^bc^ ± 0.41	1.05^g^ ± 0.05	0.92^f^ ± 0.05	0.83^g^ ± 0.03	86.20^a^ ± 0.72	398.25
Brown rice 100%	6.90^b^ ± 0.07	8.50^e^ ± 0.25	2.35^f^ ± 0.02	1.35^e^ ± 0.03	1.25^f^ ± 0.05	86.55^a^ ± 0.55	401.35
BRQ1	7.20^ab^ ± 0.08	10.70^d^ ± 0.32	2.70^e^ ± 0.06	1.65^d^ ± 0.09	1.80^e^ ± 0.07	83.15^b^ ± 0.72	399.7
BRQ2	7.40^b^ ± 0.13	11.10^cd^ ± 0.44	3.00^d^ ± 0.09	1.80^c^ ± 0.11	2.40^d^ ± 0.13	81.70^c^ ± 0.79	398.2
BRQ3	7.65^b^ ± 0.11	11.75^b^ ± 0.27	3.50^c^ ± 0.05	2.05^b^ ± 0.13	2.80^c^ ± 0.11	79.90^d^ ± 0.85	398.1
BRQ4	7.80^a^ ± 0.09	12.30^a^ ± 0.32	4.00^b^ ± 0.03	2.30^a^ ± 0.15	3.40^b^ ± 0.15	78.00^d^ ± 0.69	397.2
BRQ5	7.90^a^ ± 0.10	12.75^a^ ± 0.38	4.50^a^ ± 0.11	2.42^a^ ± 0.19	4.10^a^ ± 0.17	76.23^e^ ± 0.84	396.42
L.S.D at 0.05	0.80396	0.54598	0.12382	0.11862	0.07673	1.06002	

* Values are mean ± SD (*n* = 3). a-d Means in the same row with different superscripts are significantly different Control: 100% semolina flour; BR:100% Brown Rice; BRQ1: 90% Brown Rice with 10% quinoa flour; BRQ2: 80% Brown Rice with 20% quinoa flour; BRQ3: 70% Brown Rice with 30% quinoa flour; BRQ4: 60% Brown Rice with 40% quinoa flour; BRQ5: 50%Brown Rice with 50% quinoa flour.

### Cooking quality of pasta

Cooking losses are an important indicator of the overall pasta cooking performance for both consumers and industry, being mainly influenced by dissolving and releasing gelatinized starches from the surface of pasta through cooking water ^51^.The quality of pasta can be affected by cooking variables such as weight gain, volume gain and cooking loss. The pasta increased by 210 to 300% in weight and 160 to 215% in volume. The results showed that different blends of BRF with QF increased in weight and volume compared to the control sample (Table 7). This may be due to the protein content of QF and chickpea flour increased, leading to an increase in the weight of the cooked pasta^52^. High protein and fiber content in QF could be responsible for the improvement in cooking quality indicators. Also, the ability of the QF to absorb water and retain it in the protein–starch net. One of the most important criteria for evaluating the quality of pasta cooking is the cooking loss 19. This value indicates the amount of dry matter that is lost in the cooking water^53^. The higher the QF value, the lower the cooking losses. This result might be the result of a dough matrix reinforced by gluten and QF, which can trap starch in the resulting network; this matrix is also related to the amount of dry matter lost into the cooking water^54^. The cooking losses of different types of semolina pasta range from 6 to 11%^55^. The cooking losses for the pasta control sample, BR sample and BRQ5 sample were 2.5%, 1.65% and 1.30%, respectively. The cooking loss of the pasta samples was ≤ 8%, which is considered technologically acceptable^56^. The cooking water thickens due to the leaching of certain soluble starch and non-starch polysaccharides during the cooking of pasta^54^The results of are consistent with those of the current study^28,57^. There were significant differences in cooking loss between samples (*P* > 0.05); therefore, the addition of quinoa and chickpea at these levels affected cooking loss.

**Table 7:** Cooking quality of gluten-free brown rice pasta formulated with Quinoa flour and Chickpea flour.

Samples	Weight increase (%)	Volume increase (%)	Cooking loss (%)
Control	210^f^ ± 1.11	160^f^ ± 1.61	2.50^a^ ± 0.11
BR	235^e^ ± 1.42	170^e^ ± 1.75	1.65^b^ ± 0.15
BRQ1	250^d^ ± 2.15	180^d^ ± 1.65	1.50^c^ ± 0.17
BRQ2	270^c^ ± 2.25	190^c^ ± 1.50	1.45^cd^ ± 0.21
BRQ3	280^b^ ± 1.55	200^b^ ± 1.75	1.40^de^ ± 0.25
BRQ4	285^b^ ± 2.20	210^a^ ± 2.35	1.35^de^ ± 0.22
BRQ5	300^a^ ± 2.45	215^a^ ± 2.65	1.30^e^ ± 0.19
L.S.D at 0.05	9.52489	7.35281	0.12246

* Values are mean ± SD (*n* = 3). a-d Means in the same row with different superscripts are significantly different Control: 100% semolina flour; BR:100% Brown Rice; BRQ1: 90% Brown Rice with 10% quinoa flour; BRQ2: 80% Brown Rice with 20% quinoa flour; BRQ3: 70% Brown Rice with 30% quinoa flour; BRQ4: 60% Brown Rice with 40% quinoa flour; BRQ5: 50%Brown Rice with 50% quinoa flour.

#### Color parameters of pasta

The color of pasta is an essential indicator for determining its quality^58^. Table 8 shows a color profile of raw and cooked pasta. The dry pasta made from BRF, QF and chickpea flour was significantly darker than the dry pasta made from brown rice and dry semolina pasta (p ˂0.05). A decrease in L* values of gluten free spaghetti that underwent a cooking process. The decrease in the degree of yellowness after cooking of pasta samples could be related to leaching and thermal degradation of pigments, as already observed in legume-fortified pasta^58^. As the amount of QF in the recipe increased, the lightness (L*) of the dried pasta samples decreased. This observation is even more pronounced for pasta to which 50% more QF was added (p ˂0.05). As the amount of QF increased, the a* values decreased (greenness decreased and the redness increased). The yellow color of the quinoa variety used in our study was responsible for the large increase in b* (yellowness) in dry pasta enriched with 50 g/100 g QF. The yellow QF used in this work has an influence on this value. Compared to dry pasta, cooked pasta were characterized by greater lightness, greater greenness, and reduced yellowness. The degradation and dissolution of the pigments by hot water may be the cause of the less yellow color of the cooked pasta^59^. Regardless of the type of quinoa used, the differences in color coordinates were smaller than with dried pasta.

**Table 8:** Color quality uncooked and cooked gluten-free brown rice pasta formulated with Quinoa flour and Chickpea flour.

Pasta samples	Uncooked Pasta Sample			Cooked Pasta Sample		
	L*	a*	b*	L*	a*	b*
Control	73.02^a^ ± 1.15	4.25^a^ ± 0.05	13.85^g^ ± 0.22	78.55^a^ ± 0.97	2.20^g^ ± 0.10	18.30^a^ ± 0.32
BR	71.15^b^ ± 0.65	3.30^b^ ± 0.07	16.30^f^ ± 0.16	70.20^b^ ± 0.30	2.70^f^ ± 0.07	18.15^b^ ± 0.29
BRQ1	68.10^c^ ± 0.29	3.10^c^ ± 0.03	20.89^e^ ± 0.25	67.18^c^ ± 0.22	2.90^e^ ± 0.05	17.85^c^ ± 0.24
BRQ2	65.15^d^ ± 0.48	2.85^d^ ± 0.09	22.35^d^ ± 0.19	64.30^d^ ± 0.23	3.10^d^ ± 0.11	17.65^d^ ± 0.20
BRQ3	63.22^e^ ± 0.60	2.50^e^ ± 0.11	23.40^c^ ± 0.15	62.35^e^ ± 0.35	3.31^c^ ± 0.13	16.70^e^ ± 0.25
BRQ4	61.19^f^ ± 0.40	2.35^f^ ± 0.04	24.52^b^ ± 0.22	60.60^f^ ± 0.35	3.50^b^ ± 0.17	15.90^f^ ± 0.17
BRQ5	59.18^g^ ± 0.35	2.28^f^ ± 0.03	25.60^a^ ± 0.15	58.55^g^ ± 0.35	3.70^a^ ± 0.12	15.15^g^ ± 0.13
L.S.D at 0.05	0.10592	0.10273	0.08432	0.08797	0.06831	0.10091

* Values are mean ± SD (*n* = 3). a-d Means in the same row with different superscripts are significantly different Control: 100% semolina flour; BR:100% Brown Rice; BRQ1: 90% Brown Rice with 10% quinoa flour; BRQ2: 80% Brown Rice with 20% quinoa flour; BRQ3: 70% Brown Rice with 30% quinoa flour; BRQ4: 60% Brown Rice with 40% quinoa flour; BRQ5: 50%Brown Rice with 50% quinoa flour.

#### Sensory properties

In Table 9, the color, flavor, appearance, tenderness, stickiness and overall acceptability of pasta made from semolina(s) as control sample, BRF and BRF with QF at different concentrations (10.0, 20.0, 30.0, 40.0 and 50.0%) with 10% chickpea flour were evaluated. As shown in Table 9, consumers preferred pasta with a lower percentage of QF replacement; as the QF blend ratio increased, the pasta color decreased significantly. The color of the cooked pasta varied from 9.80 for the control followed by BR, with significant differences between them; the worst score was 6.60 for BRQ5. This result was consistent with the whiteness level (L) of the Hunter color parameter obtained, as shown in Table (8), with the amount of the dried or cooked pasta decreasing as the amount of QF increased. The values for flavor ranged from 9.65 to 6.90. BRQ5 obtained the lowest qualification with a score of 6.90. Similarly, surface stickiness was analyzed in this case, even though the scores ranged between 9.80 and 8.81, significant differences were found between all the samples. All formulations were acceptable as they received scores ranging from 48.45 to 35.21. There were significant differences in overall acceptability for all samples compared to the control (48.45). The overall acceptable results indicate that all pasta samples had good sensory scores, but the most preferable score was the pasta enriched with 10–20% QF (BRQ1-BRQ2). Notably, younger generations in particular may be more willing to accept higher levels of QF, as they are more willing to accept others and more interested in tasting new meals. Therefore, QF has a potential use in pasta applications.

**Table 9:** Sensory characteristics of cooked gluten-free brown rice paste.

Pasta samples	Color (10)	Flavor (10)	Appearance (10)	Tenderness (10)	Stickiness (10)	overall acceptability (50)
S-Control	9.80^a^ ± 0.35	9.65^a^ ± 0.31	9.60^a^ ± 0.38	9.60^a^ ± 0.54	9.80^a^ ± 0.65	48.45^a^ ± 1.19
BR	9.10^b^ ± 0.41	9.30^b^ ± 0.42	8.65^b^ ± 0.42	9.11^b^ ± 0.45	9.50^c^ ± 0.56	46.02^b^ ± 1.28
BRQ1	8.60^c^ ± 0.32	8.40^c^ ± 0.39	7.90^c^ ± 0.40	8.80^c^ ± 0. 40	9.40^d^ ± 0.48	43.10^c^ ± 1.51
BRQ2	7.70^d^ ± 0.60	8.05^d^ ± 0.51	7.55^d^ ± 0.50	7.70^d^ ± 0.50	9.15^e^ ± 0.65	40.15^d^ ± 1.32
BRQ3	7.40^e^ ± 0.50	7.80^e^ ± 0.45	7.02^e^ ± 0.60	7.10^e^ ± 0.60	9.65^b^ ± 0.63	38.97^e^ ± 1.12
BRQ4	7.00^f^ ± 0.35	7.50^f^ ± 0.52	6.60^f^ ± 0.45	6.80^f^ ± 0.53	9.17^e^ ± 0.52	37.07^f^ ± 1.22
BRQ5	6.60^g^ ± 0.28	6.90^g^ ± 0.45	6.40^g^ ± 0.35	6.50^g^ ± 0.60	8.81^f^ ± 0.52	35.21^g^ ± 1.19
L.S.D at 0.05	0.07018	0.07527	0.07769	0.06991	0.06858	0.07911

* Values are mean ± SD (*n* = 3). a-d Means in the same row with different superscripts are significantly different Control: 100% semolina flour; BR:100% Brown Rice; BRQ1: 90% Brown Rice with 10% quinoa flour; BRQ2: 80% Brown Rice with 20% quinoa flour; BRQ3: 70% Brown Rice with 30% quinoa flour; BRQ4: 60% Brown Rice with 40% quinoa flour; BRQ5: 50%Brown Rice with 50% quinoa flour.

#### Texture parameters of pasta

The texture characteristics of pasta prepared from semolina, BRF and BRF with QF at different concentrations (10, 20, 30, 40 and 50%) with 10% chickpea flour before and after cooking are shown in Tables (10, 11). The texture parameters of the pasta and the QF-enriched pasta were determined using the maximum shearing force of the sample in a Brookfield texture analyzer. The hardness (N) of the uncooked control and BR pasta samples reached 104.02 and 93.8, respectively. The amount of uncooked pasta with added QF from 10 to 50% ranged from 40.06 to 25.03 N. In contrast, pasta without QF was associated with increased effort and increased hardness due to its low moisture content. Consumers can recognize when pasta is firm, and regardless of moisture content, this could be related to the cell structure and expansion of the product. In general, consumers want pasta that is firm, chewy and non- sticky. These results are consistent with those of^60,61^. According to the texture profile results, mixing with QF at different concentrations (10 to 50%) decreased the pasta hardness (N), deformation on hardness (mm), deformation on hardness (%), hardness work (m J) and fracturability (N) with a load sensitivity of 1%. On the other hand, the hardness value decreased when the QF content in the pasta formulations increased. The matrix structure network of starch, gluten, other proteins and other ingredients has a significant effect on the texture properties of pasta^62^.

**Table 10:** Texture profile analysis of uncooked of gluten-free brown rice pasta formulated with Quinoa flour and Chickpea flour.

Pasta samples	Hardness (*N*)	Springiness Index	Chewiness Index
Control	104.02	−1.07	0.18
BR	93.80	0.18	0.20
BRQ1	40.06	0.50	0.22
BRQ2	35.60	0.60	0.45
BRQ3	30.70	0.82	0.60
BRQ4	28.50	0.92	0.75
BRQ5	25.03	1.02	0.90

Control: 100% semolina flour; BR:100% Brown Rice; BRQ1: 90% Brown Rice with 10% quinoa flour; BRQ2: 80% Brown Rice with 20% quinoa flour; BRQ3: 70% Brown Rice with 30% quinoa flour; BRQ4: 60% Brown Rice with 40% quinoa flour; BRQ5: 50%Brown Rice with 50% quinoa flour.

**Table 11:** Texture profile analysis of cooked gluten-free brown rice formulated with Quinoa flour and Chickpea flour.

	Control	BR	BRQ1	BRQ2	BRQ3	BRQ4	BRQ5
Hardness (N)	0.89	0.60	1.33	1.40	1.50	1.60	1.72
Deformation at hardness (mm)	4.25	4.40	4.60	4.70	4.90	5.05	5.35
% Deformation at hardness (%)	13.30	14.45	18.30	20.50	22.18	24.50	27.20
Hardness work (mJ)	37.00	33.00	40.00	50.00	60.00	65.00	70.00
Load at Target (N)	0.85	0.70	1.0	1.25	1.50	1.75	1.90
Peak Stress (N/m2)	51,600	44,962	48,584	55,133	57,160	59,275	61,940
Strain at Peak Load	0.18	0.16	0.19	0.22	0.25	0.27	0.30
Fracturability (N)	0.08	0.10	0.35	0.50	0.65	0.80	1.05
Fracture Load Drop Off (N)	0.02	0.01	0.03	0.05	0.07	0.09	0.10
Cohesiveness	0.35	0.22	0.42	0.44	0.47	0.52	0.55
Springiness	6.10	5.50	5.60	5.80	6.10	6.30	6.50
Gumminess	0.32	0.28	0.35	0.40	0.60	0.72	0.85
Chewiness	18.00	16.00	19.00	20.00	23.00	25.00	28.00
Adhesiveness	1.00	1.00	2.00	3.00	4.00	5.00	6.00
Resilience	0.18	0.15	0.18	0.22	0.30	0.35	0.40

Control: 100% semolina flour; BR:100% Brown Rice; BRQ1: 90% Brown Rice with 10% quinoa flour; BRQ2: 80% Brown Rice with 20% quinoa flour; BRQ3: 70% Brown Rice with 30% quinoa flour; BRQ4: 60% Brown Rice with 40% quinoa flour; BRQ5: 50%Brown Rice with 50% quinoa flour.

## Conclusions

Gluten-free pasta was developed using gluten-free, high-protein flours such as brown rice and quinoa flour. This study confirmed that the use of QF (10–50%) together with 10% chickpea flour to produce pasta was associated with acceptable nutritional, rheological, color, texture, and sensory properties. The nutritional, rheological, color and textural results of the present work demonstrate the potential of using QF as a nutritious component to produce high quality pasta. According to the judges’ total acceptance score, pasta enriched with 10% QF was acceptable in terms of sensory and textural properties. This study provides useful information for the production of gluten-free pasta products, stimulating the creative use of chickpea flour (CPF), quinoa flour (QF) and brown rice flour (BRF) as ingredients for the improvement of food security programs and the development of the food sector. Future studies could investigate the scalability of this recipe and consumer acceptance in the market. Hence Gluten free pasta can be recommended to individuals who exhibit gluten allergy or celiac disease.

## Data Availability

The datasets used and/or analyzed during the current study are available from the corresponding author upon reasonable request. I do not have any research data outside the submitted manuscript file.
